# Hepatitis virus-associated B cell non-Hodgkin’s lymphoma involves dysregulated epigenetic and RNA-mediated regulatory gene expression and altered snoRNA transcription

**DOI:** 10.1038/s41598-026-35041-3

**Published:** 2026-01-10

**Authors:** Amanda N. Henning, Myagmarjav Budeebazar, Delgerbat Boldbaatar, Dahgwahdorj Yagaanbuyant, Davaadorj Duger, Khishigjargal Batsukh, Samantha Muccilli, Jordan Pardoe, Lara Perinet, Olivia Conway, Darryl Owusu-Ansah, Kobe Robichaux, Ryan Baumann, Harvey J. Alter, Naranjargal Dashdorj, Valeria De Giorgi

**Affiliations:** 1 Department of Transfusion Medicine, Clinical Center, National Institutes of Health, Bethesda, MD USA; 2 Department of Gastroenterology, Mongolian National University of Medical Sciences, Ulaanbaatar, Mongolia; 3 Liver Center, Ulaanbaatar, Mongolia; 4 Center of Hematology and Bone Marrow Transplantation, First Central Hospital of Mongolia, Ulaanbaatar, Mongolia; 5 Onom Foundation, Ulaanbaatar, Mongolia

**Keywords:** B cell non-Hodgkin’s lymphoma (BNHL), Epigenetic regulation, ncRNA (non-coding RNA), HBV (Hepatitis B Virus), HDV (Hepatitis D Virus), snoRNA (small nucleolar RNA), Lymphoma, Hepatitis B virus, Gene expression, B cells

## Abstract

Infection with HBV and its satellite virus HDV remain a significant global health issue due to their involvement in hepatic and extrahepatic diseases, including B cell non-Hodgkin’s lymphoma (BNHL). Clinical and epidemiological evidence support a causal role for HBV in BNHL development, although mechanistic insight is lacking and the role of HDV infection in this process is unknown. To help elucidate viral drivers of B cell transformation, we performed RNA-sequencing on peripheral B cells from patients with HBV mono-infection, HBV/HDV co-infection, HBV/HDV-associated BNHL, BNHL without viral infection, and healthy donors. In this way, we sought to identify unique and shared transcriptional profiles associated with viral infection and transformation. Our data suggest dysregulated epigenetic and miRNA-mediated regulatory gene expression may be a potential common pathway for lymphomagenesis among viral- and non-viral-associated lymphoma. We also observed wide-spread upregulation of snoRNAs in B cells from virally infected patients, supporting a role for these non-coding RNAs in viral infection and, potentially, viral-associated lymphomagenesis. These results have identified novel areas for future functional studies on the effect of HBV and HDV infection on B cell activity and present additional therapeutic strategies that may benefit both viral- and non-viral associated BNHL.

**Supplementary Information:**

The online version contains supplementary material available at 10.1038/s41598-026-35041-3.

## Introduction

Globally, an estimated 254 million people are living with chronic hepatitis B virus (HBV) infection, with an estimated 4.5% co-infected with the satellite virus, hepatitis delta (HDV)^1^. HBV vaccination rates as well as HBV and HDV testing and treatment access vary by region. As a result, an estimated 87% of HBV infections go undiagnosed, and 1.2 million new HBV infections occur yearly^1^. HBV and HBV/HDV co-infection remain a significant global health issue, as chronic infection of both are well-known contributors to hepatic diseases^2,3^. Clinical and epidemiological studies also support a role for chronic HBV in the development of B cell non-Hodgkin’s lymphoma (BNHL)^3^. Chronic infection significantly increases the risk of BNHL development, especially diffuse large B cell lymphoma (DLBCL) and follicular lymphoma (FL). Further, HBV+ lymphoma patients have worse clinical outcomes than their HBV negative counterparts^3–5^. The biological mechanisms responsible for this association are unknown. Although well-established as a hepatotropic virus, there is mounting evidence supporting HBV’s lymphotropic ability. *In vivo* and *in vitro* studies have identified HBV DNA, mRNA, viral proteins, and viral integration events in bone marrow stem cells, PBMC, and mature B and T lymphocytes^3,6,7^, and HBV DNA, viral proteins, and integration events have been detected in lymphoma tissue samples from HBV-associated NHL patients^8,9^. These results suggest HBV may contribute to lymphoma development directly, through alterations stemming from B cell infection, in addition to possible indirect mechanisms. The role, if any, of HDV infection in BNHL development and its lymphotropic potential is unknown. However, HDV is dependent on HBsAg encapsulation for viral entry and exit^2^, suggesting a shared tropism between the two viruses.

Given the prevalence of HBV and HDV infection throughout the world, a deeper understanding of the specific mechanisms and cellular changes responsible for driving HBV-associated BNHL, with or without HDV co-infection, are needed. To begin to address these outstanding questions, we investigated the transcriptome of peripheral B cells from chronic HBV and HBV/HDV co-infected patients collected from Mongolia, an area of endemic HBV and HDV infection^10,11^. Our cohort consisted of patients with HBV mono-infection, HBV/HDV co-infection, HBV/HDV co-infection with BNHL, BNHL without viral infection, and healthy donors. In this way, we sought to capture early transcriptional alterations present in the periphery that may contribute to B cell transformation and lymphoma development. We report dysregulation of epigenetic and miRNA-mediated regulatory genes as a potential shared mechanism of B cell transformation for viral-associated and non-viral-associated BNHL, and upregulation of snoRNAs as a possible viral-specific mechanism. These results highlight novel areas of research that may benefit both the diagnosis and treatment of viral- and non-viral-associated BNHL and provide additional insight on the impact of HBV mono-infection and HBV/HDV co-infection on B cell activity.

## Results

### Clinical features of patient cohort

Patients were recruited for this study from the First Central Hospital of Mongolia and included individuals diagnosed with chronic HBV infection (HBV, n = 4), HBV co-infected with HDV (HBV/HDV, n = 7), co-infection of HBV and HDV with concomitant B cell non-Hodgkin’s lymphoma (BNHL/HBV/HDV, n = 3), BNHL without viral infection (BNHL, n = 6), and healthy donors (HD, n = 6). The mean age of our cohort was 47 (range 28 – 70) and 42% of participants were female (Table 1). Viral infection-only groups were younger, on average, and the HBV/HDV group had a higher proportion of male patients. This is consistent with age- and sex-specific HBV and HDV prevalence differences in the Mongolian population^10,11^. Co-infection with HDV is known to decrease HBV DNA levels^2^, and, indeed, we observed this in our cohort. Conversely, we observed no difference in HDV RNA levels among co-infected groups (Table 1, Fig. S1A,B). HBV and HDV infection are known to contribute to liver disease in chronically infected patients^2,3^. To evaluate liver functionality in our cohort, FIB-4 scores were calculated, which serve as a predictor of liver fibrosis^12^. We observed no difference in FIB-4 scores across disease groups, and none of our patients exhibited an elevated score (Fig. S1C), indicating a low likelihood of advanced fibrosis in our cohort. All patients with HBV/HDV-associated BNHL (BNHL/HBV/HDV) were classified as diffuse large B cell lymphoma (DLBCL), and the majority presented with Stage III/IV disease (67%, n = 2/3; Table 1, Fig. S1D). Our BNHL-only group was more heterogenous in subtype classification and included DLBCL (33%, n = 2), mucosa-associated lymphoid tissue (MALT) lymphoma (33%, n = 2), follicular lymphoma (FL, n = 1), and one case of undetermined subtype. The majority of BNHL-only patients (67%, n = 4/6) also presented with Stage III/IV disease (Table 1, Fig. S1D). Rates of HBV/HDV co-infection are very high in Mongolia^11^, and during the collection period we were only able to obtain one HBV-associated BNHL sample without HDV co-infection. Due to the limited sample size for this group, this sample was not included in our analyses.

**Table 1:** Patient demographics and clinical characteristics.

	Total (n = 26)	HD (n = 6)	BNHL (n = 6)	BNHL/HBV/HDV (n = 3)	HBV/HDV (n = 7)	HBV (n = 4)
Age						
Mean (SD)	47.3 (12.9)	47.7 (12.4)	54.0 (9.6)	67.3 (2.3)	38.7 (6.7)	36.5 (7.9)
Range	28–70	28–64	37–65	66–70	31–46	30–48
Female Sex, n (%)						
n (%)	11 (42)	2 (33)	4 (67)	2 (67)	1 (14)	2 (50)
HBV DNA detected						
n (%)	n/a	n/a	n/a	1 (33.3)	6 (85.7)	4 (100)
HBV DNA (log_10_ IU/mL)						
Mean (SD)	n/a	n/a	n/a	2.48 (n/a)	2.60 (1.28)	6.23 (1.19)
Range	n/a	n/a	n/a	–	1.00–4.10	4.87–7.74
HDV RNA detected						
n (%)	n/a	n/a	n/a	2 (66.7)	7 (100)	n/a
HDV RNA (log_10_ IU/mL)						
Mean (SD)	n/a	n/a	n/a	4.67 (0.56)	4.99 (2.10)	n/a
Range	n/a	n/a	n/a	4.27–5.06	1.11–7.26	n/a
BNHL Subtype^a^, n (%)						
DLBCL	n/a	n/a	2 (33.3)	3 (100)	n/a	n/a
FL	n/a	n/a	1 (16.7)	–	n/a	n/a
MALT lymphoma	n/a	n/a	2 (33.3)	–	n/a	n/a
Subtype not determined	n/a	n/a	1 (16.7)	–	n/a	n/a
Clinical stage^b^, n (%)						
Stage I	n/a	n/a	1 (16.7)	–	n/a	n/a
Stage II	n/a	n/a	1 (16.7)	1 (33.3)	n/a	n/a
Stage III	n/a	n/a	3 (50)	2 (66.7)	n/a	n/a
Stage IV	n/a	n/a	1 (16.7)	–	n/a	n/a

^a^As outlined in Revised European-American Lymphoma classification and WHO Classification of Tumors of Hematopoietic and Lymphoid Tissues (Revised 4th ed).

^b^Ann Arbor staging system.

*DLBCL* diffuse large B cell lymphoma, *FL* follicular lymphoma, *MALT* mucosa-associated lymphoid tissue.

### Transcriptional signature of immune suppression in HBV and HBV/HDV co-infected patients

**Fig. 1:**
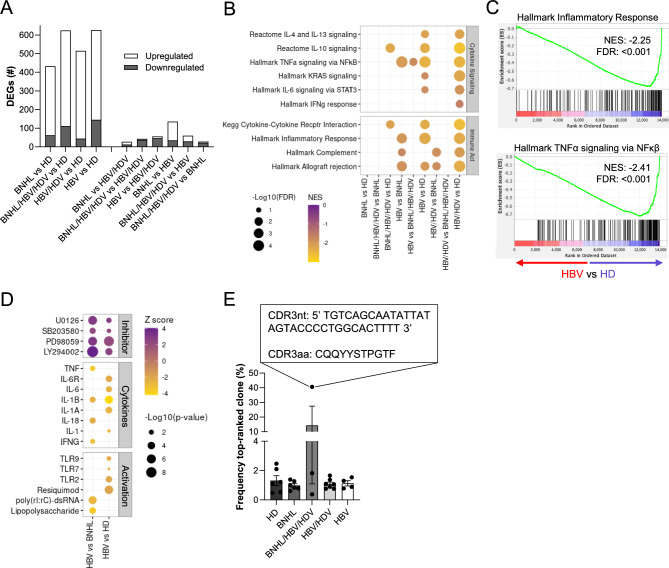
Transcriptional Analysis of Peripheral B Cells. (**A**) Stacked bar graph demonstrating the number of upregulated (white) and downregulated (gray) DEGs by comparison group. (**B**) Significantly enriched gene sets (y-axis) related to cytokine signaling and immune activation. Enrichment (NES) indicated by color and circle size denotes p-value. (**C**) Individual GSEA plots for Hallmark Inflammatory Response (top) and TNFα Signaling (bottom) gene sets in HBV vs HD. (**D**) Significantly enriched predicted upstream regulators from IPA. Enrichment Z score and p-value indicated by color and circle size, respectively. Predicted regulators are grouped based on function. (**E**) Frequency of the top-ranked clone, as defined by CDR3 nucleotide (CDR3nt) sequence. CDR3nt and amino acid (CDR3aa) sequence given for the indicated top-ranked clone. Graph displays mean ± SEM, with individual patient values indicated by circles. DEG, differentially expressed gene; FDR, false discovery rate; HD, healthy donor; NES, normalized enrichment score.

We hypothesized that investigating transcriptional changes in peripheral B cells may help elucidate the mechanisms by which HBV and HDV infection contribute to B cell transformation and BNHL development. To this end, RNA-sequencing was performed on peripheral B cells isolated from our patient cohort (HBV, n = 4; HBV/HDV, n = 7; BNHL/HBV/HDV, n = 3; BNHL, n = 6; HD, n = 6), and we performed transcriptional analysis to identify differentially expressed genes (DEGs; log2 fold change > 1 or < −1 and adjusted p-value < 0.05) among pairwise comparisons. We identified the highest number of DEGs when comparing disease groups to HDs (432–626 DEGs), with the majority upregulated relative to HDs, and we observed far fewer DEGs in comparisons between disease groups (27–135 DEGs; Fig. 1A, Table S1). Gene set enrichment analysis (GSEA) indicated a consistent negative enrichment of gene sets associated with immune activation and cytokine signaling in peripheral B cells from chronic infection-only patients relative to BNHL patients and HDs. Negative enrichment of these gene sets was much less pronounced in BNHL/HBV/HDV samples (Figs. 1B,C, S1E, Table S2). Additionally, Ingenuity Pathway Analysis (IPA) was performed to identify predicted upstream regulators of DEGs (Table S3). In HBV mono-infected samples, inhibited upstream regulators (i.e. negative Z score) included pro-inflammatory cytokines, such as TNF, IL-6, IL-1, and IFNγ, as well as known B cell activators, including multiple stimulatory molecules and toll-like receptors. Conversely, upstream regulators predicted to be activated (i.e. positive Z score) in HBV mono-infected samples included kinase inhibitors targeting important signaling pathways downstream of BCR activation, including MAPK (U0126, SB203580, and PD98059) and PI3K (LY294002)^13,14^ (Fig. 1D). Our analyses, therefore, indicate peripheral B cells from chronically infected patients exhibited a transcriptional signature of generalized immune suppression, appearing less activated and less responsive to cytokine stimulation. This trend was strongest in HBV mono-infected samples and weakest in BNHL/HBV/HDV samples. Immunosuppressive phenotypes have been observed in other immune cell types in chronic HBV infection^15^, and our data suggest peripheral B cells may be similarly impaired, although functional studies are needed to test this hypothesis.

### Investigation of anergic phenotypes in HBV/HDV-associated BNHL

In our prior study of HCV-associated BNHL^16^, we observed evidence of an anergic-like phenotype in peripheral B cells that included anergic gene expression patterns and clonal expansion. We sought to investigate whether these observations were recapitulated in HBV/HDV-associated BNHL. B cell anergy-associated gene sets^17,18^ were not significantly enriched in our BNHL/HBV/HDV samples (Fig. S1F). B cell clonal expansion was investigated using our RNA-seq data via the MiXCR analysis tool^19,20^. We observed consistency in percent and number of aligned reads, clonotype count, and predicted immunoglobulin gene usage among sample groups (Fig. S1G–J). We identified only one BNHL/HBV/HDV patient with evidence of peripheral B cell clonal expansion, with a top-ranked clonotype frequency of 40.6% (Fig. 1E). The enriched clonotype had a unique CDR3 amino acid sequence of CQQYYSTPGTF and predicted gene usage of *IGKV4-1* and *IGKJ2*. While expansion of this variable gene has been observed in HBV-associated DLBCL^21^, we did not observe wide-spread peripheral B cell expansion in our HBV/HDV-associated BNHL cohort or transcriptional evidence of B cell anergy. This is in contrast to our findings from HCV-associated BNHL patients, where peripheral clonal expansion was observed in all patients, supporting an indirect mechanism of action^16^. Our current results indicate clonal expansion may be a feature of HBV/HDV-associated BNHL, but it does not appear to be ubiquitous, and its relevance to HBV/HDV-mediated lymphomagenesis remains unclear.

### Shared transcriptional dysregulation in chronically infected and BNHL patients

**Fig. 2:**
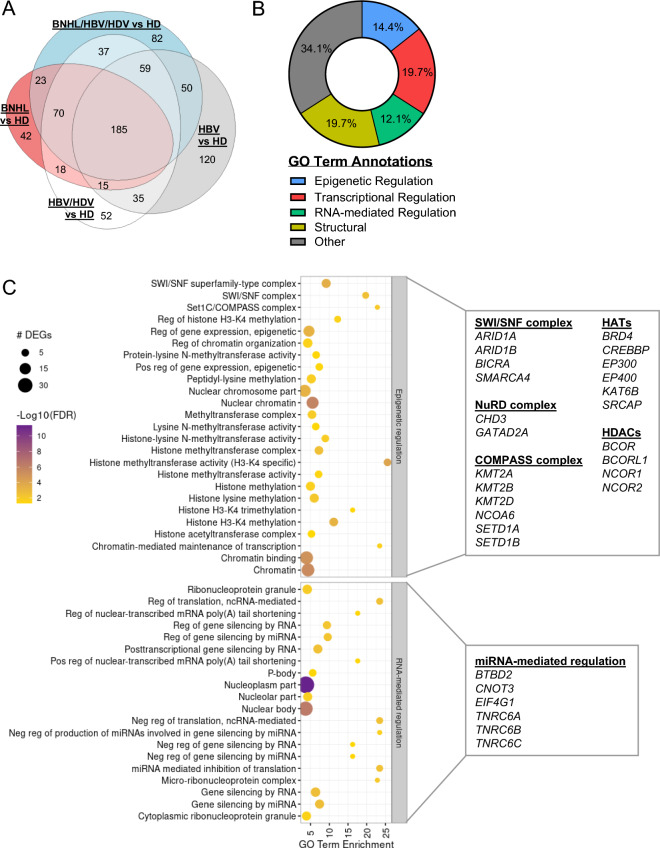
Shared Transcriptional Dysregulation in Chronically Infected and BNHL Patients. (**A**) Area proportional Venn diagram illustrating the overlap of upregulated DEGs between indicated comparison groups. (**B**) Summary of manual annotation of significant GO terms (n = 223) identified by GO analysis of n = 185 commonly upregulated genes across disease groups vs HDs. (**C**) Most significantly enriched GO terms from the Epigenetic Regulation and RNA-mediated Regulation groups referenced in B are listed (y-axis). GO term enrichment shown on the x-axis, p-value indicated by color, and the number of DEGs annotated to each GO term indicated by circle size. Differentially expressed genes of interest are listed and grouped by function/complex.

Most DEGs were found to be upregulated in peripheral B cells from disease groups when compared to HDs (Fig. 1A), and we sought to further investigate these upregulated genes to uncover transcriptional commonalities across disease states. We observed 185 genes to be significantly upregulated in HBV, HBV/HDV, BNHL/HBV/HDV, and BNHL samples relative to HDs (Fig. 2A). Gene ontology (GO) analysis of these genes revealed enrichment of GO terms related to epigenetic and transcriptional regulation, cytoskeleton structure, and RNA-mediated regulation (Fig. 2B, Table S4). Specifically, enriched epigenetic GO terms included broad terms related to chromatin organization and histone methylation, as well as regulatory complex-specific terms (Fig. 2C). Indeed, upregulated genes included several known components of the SWI/SNF and NuRD chromatin remodeling complexes, including *ARID1A/B* and *GATAD2A*, respectively, as well as members of the COMPASS histone methyltransferase complex, including *KMT2A/B/D* and *SETD1A/B*. Additionally, several well-known histone acetyltransferases (*BRD4*, *CREBBP*, *EP300*) and histone deacetylases (*BCOR*, *NCOR1/2*) were upregulated (Fig. 2C). GO terms related to RNA-mediated regulation were highly enriched, and annotated genes included critical components facilitating miRNA-mediated gene silencing, including members of the RISC complex (*TNRC6A*, *TNRC6B*, and *TNRC6C*)^22^, *CNOT3*, a component of the mRNA deadenylase CCR4-NOT complex^22^, and the translation initiation factor *EIF4G1*^23^ (Fig. 2C). Peripheral B cells from chronically infected patients, therefore, shared a transcriptional phenotype of dysregulated epigenetic and miRNA-mediated regulatory gene expression with B cells from BNHL patients, suggesting these regulatory pathways may be involved in both viral- and non-viral-associated lymphomagenesis.

### Elevated snoRNA expression as a possible viral-specific mechanism of lymphomagenesis

**Fig. 3:**
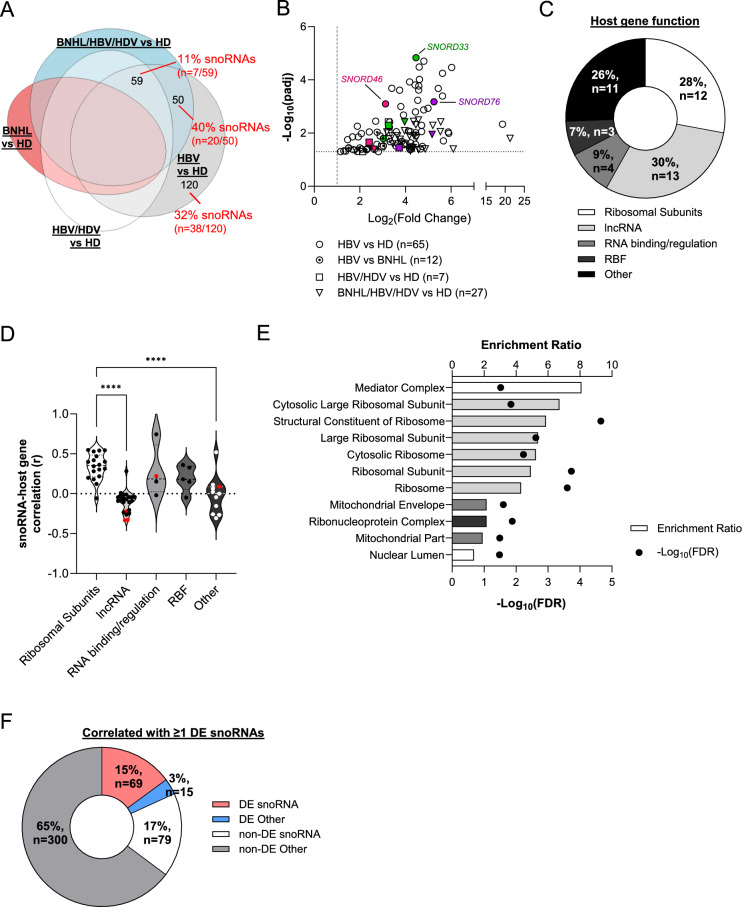
Elevated snoRNA Expression as a Possible Viral-specific Mechanism of Lymphomagenesis. (**A**) Area proportional Venn diagram highlighting the number of differentially expressed (DE) snoRNAs among chronically infected groups. (**B**) Volcano plot of upregulated DE snoRNAs. Comparison groups with the highest number of DE snoRNAs are shown. Number of snoRNAs per group indicated in legend. Fold change and p-value cutoffs for differential expression indicated on x-axis and y-axis, respectively (dashed and dotted lines). Specific snoRNAs of interest are labeled and indicated with colored shapes. (**C**) Manual classification of DE snoRNA host gene function. (**D**) Violin plot of gene expression correlation (Pearson r) between DE snoRNAs and their corresponding host gene. Host genes are grouped by their function. Red circles indicate snoRNA-host gene pairs where the host gene is also differentially expressed. Plots show median (dashed line) and 25^th^ and 75^th^ percentiles (dotted lines). (**E**) Significantly enriched GO terms from transcriptome-wide analysis of genes whose expression highly correlates (Pearson r > 0.7) with at least one DE snoRNA. GO terms listed on the y-axis and color coded based on related function. Bars indicate enrichment ratio (top x-axis), and circles indicate p-value (bottom x-axis). (**F**) DE status of genes correlated with at least one of the DE snoRNAs identified in our study. DE, differentially expressed; FDR, false discovery rate; HD, healthy donor; lncRNA, long non-coding RNA; RBF, ribosome biogenesis factor. *p* < 0.0001, ****.

In addition to identifying shared pathways among disease groups, we were interested in dysregulated pathways that may be unique to chronic hepatitis infection. We observed an enrichment of small nucleolar RNAs (snoRNAs) among upregulated genes in chronically infected groups relative to healthy donors (Fig. 3A). Across all comparison groups, we observed 69 unique snoRNAs to be differentially expressed (DE), the majority of which were upregulated in B cell samples from HBV mono-infection patients relative to HDs (n = 65) (Fig. 3B, Table S1). snoRNAs are a type of small non-coding RNA, whose canonical function is to aid in ribosome biogenesis within the nucleolus by directing post-transcriptional nucleotide modifications of ribosomal RNA (rRNA)^24^. Importantly, viruses, including HBV and HDV, are known to hijack aspects of nucleolar activity, including ribosomal biogenesis, to promote viral replication and translation^25,26^. Traditionally, snoRNAs are encoded in intronic regions of protein-coding and non-coding host genes, and the gene products of protein coding snoRNA host genes tend to be involved in ribosome biogenesis, regulation of translation, or RNA processing^27^. We observed this to be the case for our DE snoRNAs. Almost all DE snoRNAs (n = 68/69) were intronically located, and host genes consisted of long-noncoding RNAs (lncRNAs; 30%), ribosomal subunits (28%), ribosome biogenesis factors (RBF, 7%), or encoded proteins involved in RNA binding/regulation (9%) (Fig. 3C). Despite their intragenic location, snoRNA expression does not always correlate with host gene expression^27,28^. Fafard-Couture et al.^28^ observed that snoRNAs whose expression was positively correlated to their host genes tended to reside within ribosomal protein genes. Indeed, in our cohort, correlation values (Pearson correlation coefficient, r) for snoRNA-host gene pairs were elevated for host genes encoding ribosomal subunits compared to host genes with other functions (Fig. 3D). Additionally, despite differential expression of their intronic snoRNA, only three host genes were found to be differentially expressed, corresponding to five DE snoRNA-host gene pairs (Fig. 3D). Together, this indicates that our observed snoRNA differential expression is not solely driven by host gene expression differences. We expanded our correlation analysis to include all expressed genes and observed 463 genes with high positive correlation with at least one DE snoRNA (Pearson r > 0.7). GO analysis of this correlated gene set indicated enrichment of Mediator complex and ribonucleoprotein genes as well as ribosomal subunits (Fig. 3E). Although the majority of correlated genes were not differentially expressed (Fig. 3F), correlation analysis suggests possible transcriptional co-regulation of DE snoRNAs with numerous genes involved in ribosome biogenesis. In this way, upregulation of snoRNA genes in peripheral B cells from chronically infected patients may indicate infection-dependent alterations in ribosomal biogenesis or composition.

snoRNAs have gained increasing interest for their role in cancer detection and progression, with numerous reports of specific snoRNAs serving as diagnostic and/or prognostic biomarkers and as having oncogenic and tumor suppressive roles^24^. Often, effects on cancer development are driven by non-canonical snoRNA functions that can include direct regulation of mRNA expression at the post-transcriptional or translational level^24,29^. Of our DE snoRNAs, we found 31 that had documented roles in cancer or mRNA regulation (Table 2). This included three snoRNAs that were upregulated in all chronically infected groups relative to HDs (*SNORD33*, *SNORD46*, and *SNORD76*; Fig. 3B). Cancer-associated roles were reported across several histologies, and many snoRNAs appeared to have cancer type-specific effects. For instance, functional studies support an oncogenic role for *SNORD76* in hepatocellular carcinoma (HCC) and a tumor suppressive role in glioblastoma, where it acts to either promote or inhibit cell proliferation, respectively^30,31^. Overall, our transcriptional analysis indicated elevated snoRNA expression may be a viral-specific phenomenon in peripheral B cells, and it presents the intriguing hypothesis that snoRNA-mediated alterations to ribosomal biogenesis or mRNA transcription and translation may be a mechanism driving viral-associated lymphomagenesis.

**Table 2:** Role of differentially expressed snoRNAs in cancer and post-transcriptional regulation.

snoRNA function	Differentially expressed snoRNA(s)
Cancer biomarker (diagnostic and/or prognostic)	
AML	*SNORA31*^56^
BC	*SNORA10*^57^, *SNORA24*^57^, *SNORA52*^57^, *SNORA66*^58^, *SNORD33*^59^, *SNORD46*^60^, *SNORD89*^60^
CRC	*SNORD16*^61^, *SNORD57*^62^, *SNORD67*^63^
DLBCL	*SNORA60*^64^, *SNORA66*^64^
HCC	*SNORA52*^65^, *SNORD46*^66^, *SNORD76*^30^
MM	*SNORD27*^67^
NSCLC	*SNORD104*^68^, *SNORD28*^69^, *SNORD33*^70^, *SNORD55*^71^, *SNORD60*^72^, *SNORD66*^70^, *SNORD76*^70^, *SNORD83A*^73^
OC	*SNORD89*^74^
PC	*SNORA14B*^75^
PCa	*SNORA10*^76^
RCC	*SNORD35B*^77^, *SNORD60*^77^
SKCM/UM	*SNORA31*^78^, *SNORD12*^79^, *SNORD83A*^78^
Oncogenic activity	
BC	*SNORD28*^48^, *SNORD46*^80^, *SNORD50A*^47^
CRC	*SNORA24*^46^, *SNORD16*^61^
EC	*SNORD104*^50^, *SNORD60*^51^, *SNORD89*^49^
HCC	*SNORD76*^30^
NSCLC	*SNORA7A*^81^, *SNORD46*^80^
OC	*SNORA81*^82^, *SNORD89*^74^
OS	*SNORA7A*^83^
PC	*SNORD35A*^84^
Tumor suppressive activity	
GBM	*SNORD47*^85^, *SNORD76*^31^
HCC	*SNORA24*^86^
KRAS-driven cancers	*SNORD50A*^87^
Regulation of mRNA splicing and processing	*SCARNA1*^88^, *SNORD27*^89^, *SNORD50A*^90^, *SNORD83B*^91^
Effect on ribosome heterogeneity	*SNORA33*^92^

*AML* acute myeloid leukemia, *BC* breast cancer, *CRC* colorectal cancer, *DLBCL* diffuse large B cell lymphoma, *EC* endometrial cancer, *GBM* glioblastoma, *HCC* hepatocellular carcinoma, *MM* multiple myeloma, *NSCLC* non-small cell lung cancer, *OC* ovarian cancer, *OS* osteosarcoma, *PC* pancreatic cancer, *PCa* prostate cancer, *RCC* renal cell carcinoma, *SKCM* human skin cutaneous melanoma, *UV* uveal melanoma.

## Discussion

Infection with HBV and its satellite virus HDV remain a significant global health issue, with chronic infection contributing to increased risks of hepatic and extrahepatic diseases, including B cell non-Hodgkin’s lymphoma (BNHL)^3^. Clinical and epidemiological evidence support a role for chronic HBV in BNHL development^3^. However, the precise mechanisms by which HBV may contribute to lymphomagenesis and the contributions of chronic HDV infection are unclear. To begin to address these gaps in knowledge, we sought to investigate the peripheral B cell transcriptome of HBV/HDV-associated BNHL in relation to non-viral associated BNHL as well as chronic infection alone and healthy donors. By analyzing circulating B cells from chronically infected patients both with and without lymphoma, we hoped to identify early transcriptional changes that may contribute to lymphomagenesis via either direct or indirect mechanisms.

**Fig. 4:**
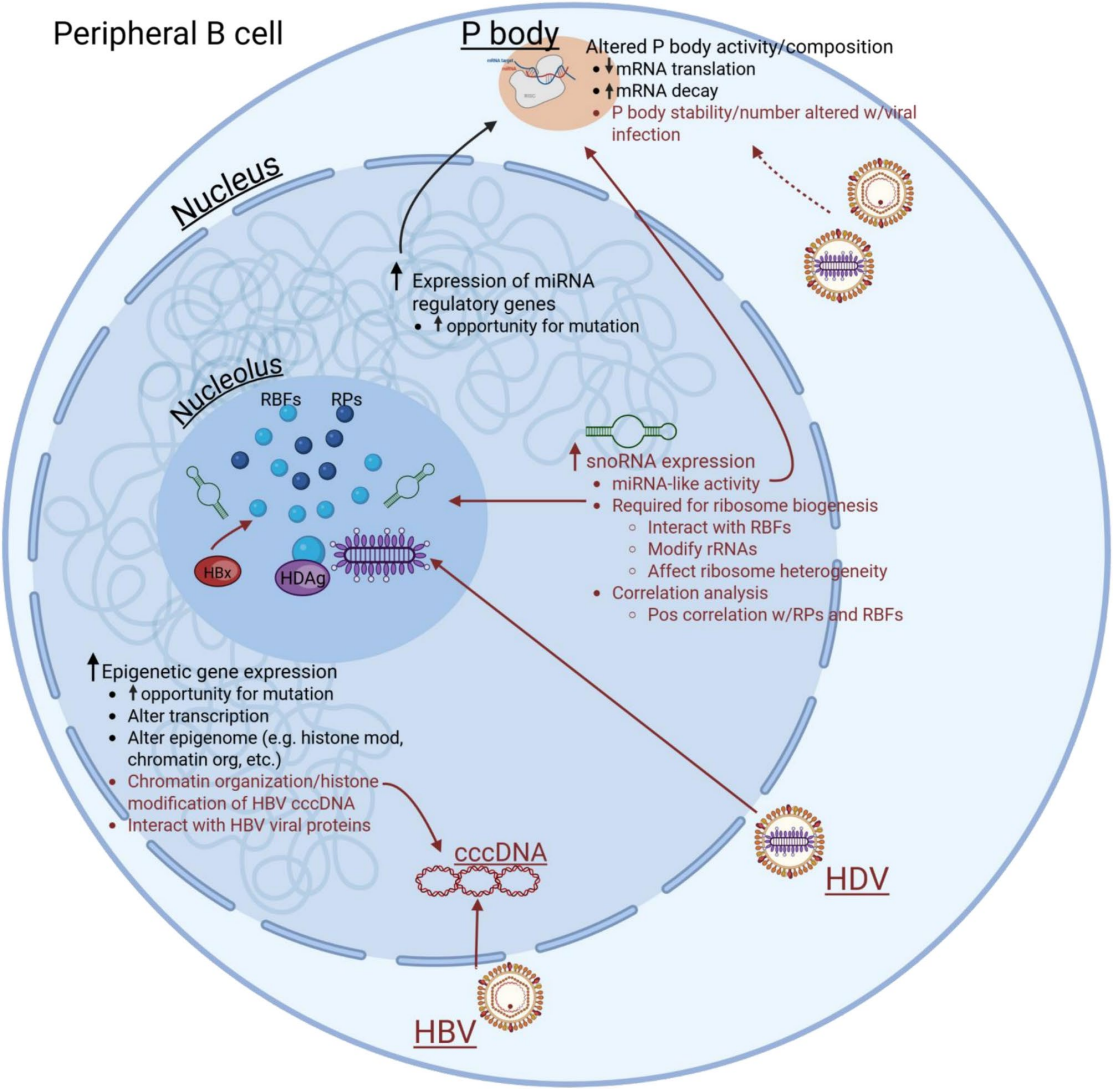
Proposed Direct Mechanisms of HBV and HBV/HDV-associated B cell Transformation. Based on our transcriptional analyses, we illustrate possible direct mechanisms by which HBV and HDV may be contributing to B cell transformation in a lymphotropic model of infection. Black text indicates dysregulated pathways that may be shared between viral- and non-viral-associated BNHL, and red text indicates dysregulated pathways and interactions that may be unique to HBV and HDV infection. Dashed line indicates hypothesized interaction warranting further investigation. Figure created using BioRender (Henning, A. (2025) https://BioRender.com/5k2rgb3). cccDNA, covalently closed circular DNA; HBx, HBV-encoded oncogene X protein; HDAg, hepatitis delta antigen; miRNA, micro RNA; mRNA, messenger RNA; P body, processing body; RBF, ribosome biogenesis factor; RISC, RNA-induced silencing complex; RP, ribosomal protein; rRNA, ribosomal RNA; snoRNA, small nucleolar RNA.

Differential expression analysis revealed that all disease states exhibited upregulation of epigenetic regulatory genes and genes required for miRNA-mediated gene silencing. Tightly controlled epigenetic regulation is an essential component of normal B cell development^32^, and disruption of these regulatory pathways is frequently observed in lymphomas^33^. Indeed, epigenetic regulators are frequently mutated in BNHL^33^, including many of our DEGs, such as *KMT2D*, *CREBBP*, *EP300*, and *ARID1A*, which are each individually found to be mutated in approximately 5 – 25% of non-viral and HBV-associated DLBCL cases^5,34–36^. Epigenetic regulatory proteins are also known to be affected by viral infection, including chronic HBV. In hepatocytes, the HBV viral genome translocates to the nucleus where it utilizes host histones and epigenetic modifying proteins to establish covalently closed circular DNA (cccDNA), which is required for efficient viral transcription and replication. The viral protein HBx is known to aid in this process and has been shown to directly interact with host epigenetic proteins, including p300/*EP300* and CBP/*CREBBP*^37,38^. In this way, the upregulation of epigenetic modifiers we observed in peripheral B cells may aid in HBV replication in a lymphotropic model of infection, although functional studies would be needed to test this hypothesis. miRNA-mediated regulation of gene expression represents another regulatory mechanism employed during normal B cell development and activation whose dysregulation contributes to B cell transformation^32^. We observed upregulation of multiple genes encoding proteins required for miRNA-mediated mRNA regulation. In particular, *TNRC6A*, *TNRC6B*, and *TNRC6C* form a critical component of the RISC complex, which facilitates miRNA-mediated mRNA decay and translational repression. This regulatory activity occurs within membraneless, cytoplasmic domains called processing bodies (P bodies)^22^. Alterations to P body activity and composition have been implicated in the development of multiple carcinoma subtypes and represents a novel diagnostic marker and therapeutic target^39^. Additionally, P body stability, number, and localization have been shown to be altered following viral infection^40,41^, although it is unknown whether HBV or HDV infection affect their activity. Our results support additional studies on the specific role of P bodies in B cell biology, transformation, and chronic hepatitis infection. Altogether, the upregulation of epigenetic modifiers and RNA-dependent regulatory genes we observed in viral- and non-viral-associated BNHL and chronic infection would not only increase the opportunity for loss- or gain-of-function mutations at these loci^42^, but could alter the transcriptomic, epigenomic, and/or translational landscape of circulating B cells (Fig. 4). In this way, these gene expression changes may be a shared molecular response contributing to a pro-lymphomagenic cellular environment and represent a potential common pathway driving viral and non-viral lymphomagenesis.

Despite compelling clinical evidence supporting the role of HBV in BNHL development, there is very little information on the mechanisms behind this association or the functional effects of chronic infection on B cell activity. Furthermore, there is virtually no information on the impact of HBV/HDV co-infection. Our transcriptional profiling has identified multiple biological programs that appear dysregulated in peripheral B cells during chronic infection with HBV and HBV/HDV that may be contributing to viral-associated BNHL development (Fig. 4). In particular, we observed upregulation of numerous snoRNAs in B cells from chronically infected patients, especially HBV mono-infected patients. Canonical snoRNA activity occurs within the nucleolus, where they interact with ribosome biogenesis factors (RBFs) to add 2′-O-methylation or pseudouridylation modifications to rRNAs, facilitating ribosome biogenesis^24^. We observed differentially expressed snoRNAs to be positively correlated with RBF and ribosomal protein gene expression. This suggests possible co-regulation of these genes and supports the hypothesis that chronic HBV/HDV co-infection and HBV mono-infection may result in wide-spread alterations to ribosome biogenesis in peripheral B cells. HBV and HDV are known to commandeer nucleolar activity, including ribosome biogenesis, to support viral replication and translation^25,26^. In a lymphotropic model of infection, this has the potential to promote B cell transformation. Indeed, in HBV-associated HCC, HBx was found to interact with the RBF nucleophosmin in the nucleolus to promote ribosome biogenesis, resulting in increased cell proliferation and transformation^43^. Furthermore, replication of HDV genomic RNA occurs in the nucleolus, where HDAg interacts with RBFs to initiate antigenomic RNA synthesis needed for viral propagation^44,45^. snoRNAs have also been shown to have direct roles in cancer development^24^, and many of our differentially expressed snoRNAs have demonstrated oncogenic activity (Table 2). *SNORA24*, *SNORD28*, and *SNORD50A* have been shown to negatively regulate p53 protein stability in colorectal cancer and breast cancer^46–48^. *SNORD28* promotes oncogenesis via miRNA-like activity directed against the p53-stabilizing protein TAF9B^48^, while *SNORD50A* promotes the ubiquitination and subsequent cytoplasmic sequestration of the p53-stabilizing protein GMPS^47^. In endometrial cancer, *SNORD60*, *SNORD89*, and *SNORD104* promote tumorigenesis by directing their 2′-O-methylation activity towards protein-coding mRNAs. This post-transcriptional modification affects the stability or translation efficiency of target mRNAs, leading to alterations in downstream signaling^49–51^. While our current study cannot determine if snoRNA expression changes are due to indirect or direct effects of chronic infection, the known nucleolar activity of HBV and HDV together with their reported lymphotropic potential support the intriguing hypothesis that snoRNA upregulation may be a direct mechanism contributing to viral-mediated B cell transformation and represents a novel area of future investigation.

In the current study, we have identified a number of specific biological pathways that may be contributing to HBV and HBV/HDV-associated BNHL development. Our identification of P bodies as possible effectors of downstream alterations in both viral- and non-viral associated BNHL represents a novel finding with therapeutic implications and warrants additional investigation. Additionally, viral-specific snoRNA upregulation represents a possible novel mechanism contributing to B cell transformation at the chronic infection stage, and it is consistent with known relationships between HBV and HDV infection and nucleolar activity. We cannot, however, determine whether viral-specific alterations are the result of indirect mechanisms that are occurring as a cellular response to viral infection or are initiated via direct mechanisms following viral infection of B cells. In HBV-associated lymphomas, evidence of viral integration events, viral-specific mutational signatures, and inconsistent reports of biased immunoglobulin gene usage support direct mechanisms as the primary driver of HBV-mediated B cell transformation^4,5,8^. However, while evidence supports the lymphotropic potential of HBV^3,6,7^, it is still an ongoing area of research in the field. The RNA-sequencing method used in this study was not designed to detect viral transcripts in patient B cells, and our limited amount of patient material prevented viral-specific sequencing for all patients. However, we were able to perform unbiased RNA-sequencing on one HBV+ mono-infected patient and were able to detect HBV transcripts with high sequence identity (> 90%) and complete genome coverage (Fig. S2). While this finding warrants additional investigation in a larger cohort, it further supports HBV’s lymphotropic potential and the opportunity for B cell transformation via direct mechanisms. This does not, however, exclude the possibility that indirect mechanisms may also be employed. In particular, extracellular vesicles can facilitate HBV propagation and crosstalk between hepatocytes and lymphocytes, disseminating not only viral DNA and proteins, but host proteins and RNAs that can mediate functional changes in recipient cells to support disease progression^52^. Caveats to our study include a limited sample size and subtype heterogeneity in the BNHL-only cohort. Additionally, we were unable to obtain HBV-associated BNHL samples without HDV co-infection, and our viral-only group skewed younger. Despite these limitations, our results support novel hypotheses regarding the mechanisms underlying the epidemiological association between chronic hepatitis infection and BNHL development. Specifically, we identified epigenetic and miRNA-mediated regulation as possible effectors of downstream alterations in both viral- and non-viral associated BNHL and snoRNA upregulation as a possible novel viral-specific mechanism contributing to B cell transformation. These data begin to shed light on an understudied area in the field and support additional, functional studies that will have significant diagnostic and therapeutic implications for patients with both chronic infection and lymphoma.

## Methods

### Patient population and clinical testing

Patients were recruited for this study at the First Central Hospital of Mongolia (Ulaanbaatar, Mongolia) according to study protocol policies approved by the Ethics Committee of the Ministry of Health (No. 4, approval date 6/19/2017). Informed consent was obtained from all patients, and all methods were performed in accordance with relevant guidelines and regulations. BNHL diagnosis was determined using cytological or pathological examination and guidelines outlined in the Revised European-American Lymphoma classification^53^ and the WHO Classification of Tumors of Hematopoietic and Lymphoid Tissues (Revised 4th edition)^54^. Clinical blood tests were performed at the First Central Hospital of Mongolia, and viral testing took place at the Liver Center (Ulaanbaatar, Mongolia). HBV DNA and HCV RNA testing was performed using the Xpert® HBV and HCV Viral Load assays, respectively, run on the automated, closed-system GeneXpert® IV or XVI platform (Cepheid; Sunnyvale, CA, USA). Anti-HCV antibody detection was conducted via the HCV Ab Plus Rapid Test (CTK Biotech; Poway, CA, USA). Samples testing positive were subject to HCV RNA quantitative testing. Qualitative testing of HBsAg was conducted using the HBsAg Rapid test (CTK Biotech; Poway, CA, USA). HDV RNA quantification was conducted using an in-house developed assay^55^. The FIB-4 scoring metric was used to estimate liver fibrosis in patients using the formula (Age*AST)/(Platelets*sqrt(ALT)). Patients with FIB-4 scores above 3.25 are likely to suffer from advanced fibrosis, while patients with scores below 1.45 are likely free from fibrosis^12^.

### PBMC sample processing, B cell isolation and RNA-sequencing

Whole blood collection and PBMC isolation was performed by collaborators in Mongolia at the Liver Center as outlined in Henning et al.^16^. B cell isolation, RNA isolation, and RNA-sequencing was performed as previously described^16^. Briefly, B cells were isolated from thawed PBMC samples via the EasySep Human B cell Isolation kit (StemCell Tech) and RNA was isolated using the RNeasy Plus Micro kit (Qiagen). The TruSeq RNA Exome kit (Illumina) was used for library preparation, and RNA-sequencing was performed on a NextSeq 550 instrument (Illumina) using High Output flow cells (v2.5, 150 cycles; Illumina).

### Bioinformatic and statistical analysis

The BaseSpace Sequencing Hub (Illumina) was used to evaluate sequencing run quality metrics and for alignment. Reads were aligned using the RNA-Seq Alignment application (v2.0.2, hg19 reference genome), which utilizes the STAR aligner and Salmon for transcript quantification. Sequencing files have been deposited in the GEO repository under GSE279755. BCR repertoire analysis using RNA-seq input data was conducted using the MiXCR Immune Repertoire Analyzer application (v2.1.11)^19,20^. Differential expression analysis was performed in R (v4.0.2) using the DESeq2 package (v1.28.1; DESeqDataSet design = ~ Batch + Diagnosis), and differentially expressed genes (DEGs) were defined as those with a log2 fold change < −1 or > 1 and a Benjamini–Hochberg adjusted P-value of < 0.05. Bioinformatic tools used for transcriptional investigations included: gene set enrichment analysis (GSEA, v4.0.3), Ingenuity Pathway Analysis (IPA, Qiagen), and gene ontology analysis (WebGestalt tool, www.webgestalt.org). Transcriptome-wide correlation (Pearson correlation coefficient, r) was performed in R, and figures were created using R and GraphPad Prism (v10.1.2). Statistical analysis was performed in GraphPad. An unpaired T test or nonparametric Mann–Whitney test was used for analyses consisting of 2 groups. For groups of more than 2, a one-way ANOVA or nonparametric Kruskal–Wallis test was performed, with Tukey’s or Dunn’s correction for multiple comparisons, respectively.

## Supplementary Information

Supplementary Information 1.

Supplementary Information 2.

Supplementary Information 3.

Supplementary Information 4.

Supplementary Information 5.

Supplementary Information 6.

## Data Availability

The datasets generated and analyzed during the current study are available in the GEO repository, [https://www.ncbi.nlm.nih.gov/geo/](https:/www.ncbi.nlm.nih.gov/geo), under accession number GSE279755. Additional information and files are available on reasonable request.
