# Supplementary material for: Hepatitis virus-associated B cell non-Hodgkin’s lymphoma involves dysregulated epigenetic and RNA-mediated regulatory gene expression and altered snoRNA transcription
Source: Sci Rep. 2026 Jan 10;16:5003. doi: 10.1038/s41598-026-35041-3 (PMC12876061; doi:10.1038/s41598-026-35041-3)
Supplement: Supplementary file 1 — Supplementary Information 1. [file 41598_2026_35041_MOESM1_ESM.pdf]

Supplemental Figure 1

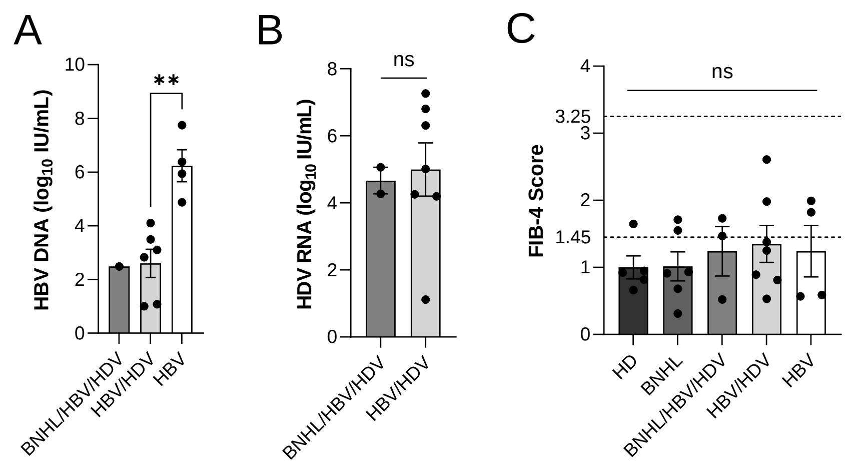

D

Disease Group	BNHL Subtype	Lymphoma stage (Ann-Arbor) 1	2	3	4
BNHL	DLBCL			1	1
	FL			1	
	MALT lymphoma		1	1	
	Not determined	1			
BNHL/HBV/HDV	DLBCL		1	2	

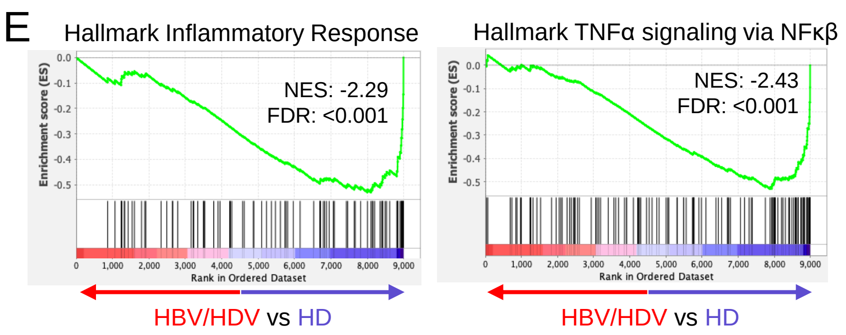

F

BNHL/HBV/HDV vs HD

	Size	NES	FDR
ISNARDI_CD21lo_UP	48	-0.68	1.000
ISNARDI_CD21lo_DN	25	-0.45	0.992
CHARLES_MC_UP	32	1.30	0.150
CHARLES_MC_DN	29	-1.12	0.289

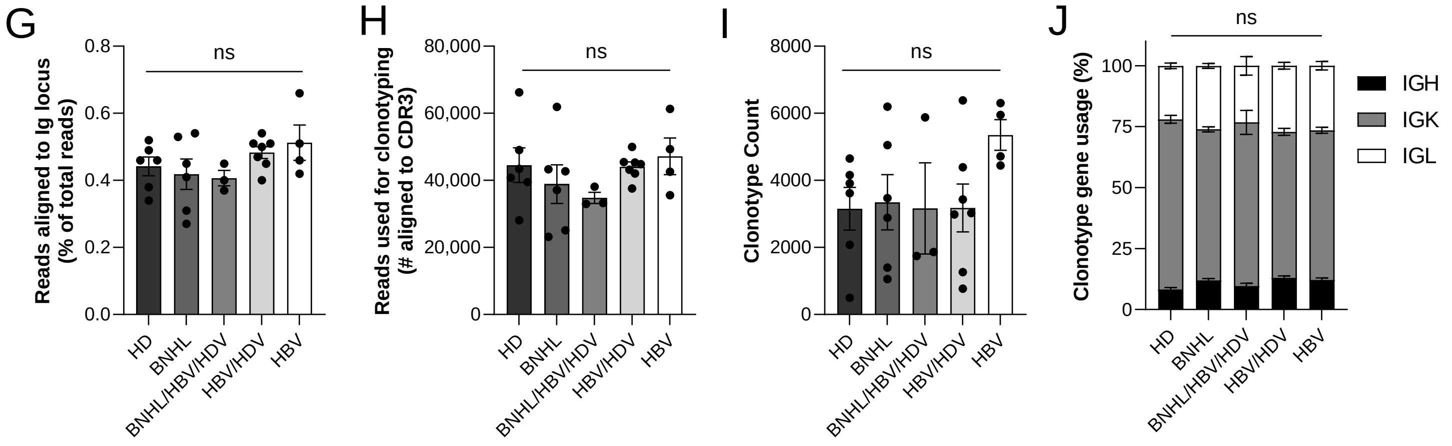

Supplemental Figure 1 – Transcriptional Analysis of Peripheral B Cells. A-B) Quantification of HBV DNA (A) and HDV RNA (B) levels in patient samples. **C)** FIB-4 scores were calculated according to the formula (Age*AST)/(Platelets*√ALT). A score <1.45 suggests an absence of advanced fibrosis, while a score >3.25 suggests the presence of advanced disease (dashed lines). **D)** Breakdown of lymphoma subtypes and stage in BNHL and BNHL/HBV/HDV groups. **E)** Individual GSEA plots for Hallmark Inflammatory Response (left) and TNFα Signaling (right) gene sets in HBV/HDV vs HD. **F)** GSEA results of BNHL/HBV/HDV vs HD comparison for published gene sets found to be upregulated (UP gene sets) or downregulated (DN gene sets) in anergic B cells [Ref: Isnardi *et al*, *Blood*, 2010; Charles *et al*, *Blood*, 2011]. **G-I)** Graphs display percent of Ig aligned reads (G), number of reads used for clonotyping (H), and clonotype count (I) across samples. **J)** Stacked graph indicates the percentage of identified clones corresponding to heavy (IGH) or light (IGK and IGL) chain genes. All bar graphs display mean ±SEM, with individual patient values indicated by circles. DLBCL, diffuse large B cell lymphoma; FDR, false discovery rate; FL, follicular lymphoma; HD, healthy donor; MALT, mucosa-associated lymphoid tissue; NES, normalized enrichment score; ns, not significant. $p<0.01$, **.
