# Supplementary material for: Hepatitis virus-associated B cell non-Hodgkin’s lymphoma involves dysregulated epigenetic and RNA-mediated regulatory gene expression and altered snoRNA transcription
Source: Sci Rep. 2026 Jan 10;16:5003. doi: 10.1038/s41598-026-35041-3 (PMC12876061; doi:10.1038/s41598-026-35041-3)
Supplement: Supplementary file 2 — Supplementary Information 2. [file 41598_2026_35041_MOESM2_ESM.pdf]

Supplemental Figure 2

A

	# of Reads	% of Total Input
Total input	286,565,338	100%
After QC	276,247,154	96.4%
After filtering out non-viral (host) reads	5,892,674	2.1%
Mapped to contigs	4,536,046	1.6%

B

Viral Assignment	# of Reads	Depth of Coverage	NT Identity	AA Identity	Genome Coverage
Hepatitis B virus	12,514	282.0	90.3%	92.8%	100.0%

C

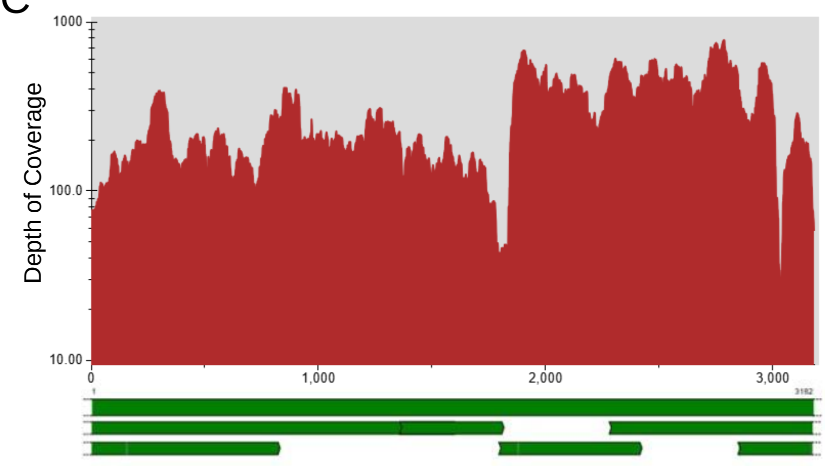

Supplemental Figure 2 – HBV Detection in Peripheral B Cell RNA from HBV+ patient. We performed additional RNA-sequencing on B cell RNA from an HBV+ mono-infection patient to investigate the presence of HBV viral transcripts. 80ng of input RNA was available for library preparation using the Illumina Stranded Total RNA Prep with Ribo-Zero Plus kit, and sequencing was performed on a NextSeq 550 instrument using a high output flow cell (v2.5, 150 cycles; Illumina). FASTQ files were uploaded to the web-based software application Genome Detective (genomedetective.com; v2.21.3), which performs filtering, trimming, pan-viral alignment, and viral identification (Vilsker *et al*, *Bioinformatics*, 2019; PMID: 30124794). **A**) Sequencing metrics for reads passing quality control (QC) and viral alignment. **B**) Summary table of the identification of HBV reads which includes the total number of reads, estimated depth of coverage, percent nucleotide (NT) and amino acid (AA) identity (HBV reference genome NC_003977.2), and genome coverage. **C**) Coverage map illustrating the depth of coverage across the HBV genome (top) as well as a depiction of the genome region and codon alignment (bottom).
